# Advanced hybrid closed-loop system: first successful clinical case after total pancreatectomy

**DOI:** 10.1007/s00592-021-01715-9

**Published:** 2021-04-16

**Authors:** A. Rizzi, L. Tartaglione, M. Di Leo, S. Alfieri, D. Pitocco

**Affiliations:** 1 Diabetes Care Unit, Catholic University, Universitario Agostino Gemelli, L.Go Agostino Gemelli, 800168 PoliclinicoRome, Italy; 2 Surgery Department, Catholic University, Universitario Agostino Gemelli, PoliclinicoRome, Italy

## Main Text

A 64-year-old woman has undergone in February 2019 total spleen-preserving pancreatectomy for cystic pancreatic neoplasia. In her medical history, in 2010 she also underwent total thyroidectomy because of thyroid cancer. She is a former smoker who quitted smoking in 2014. From February 2019, she assumes pancrelipase 10.000 UI daily as pancreatic replacement therapy and from 2010 levotiroxine for thyroid replacement.

At the discharge, insulin therapy with multiple daily injections, supported by advanced educational therapeutic plan about carbohydrates counting, was started, but, after a severe hypoglycemic event, she developed an important fear of hypoglycemia with a consequent wrong approach to the insulin therapy, preferring to maintain glycemic values higher than 200 mg/dL in order to avoid hypoglycemia. Insulin therapy with continuous subcutaneous insulin infusion (CSII) was suggested, but she refused mainly because of discomfort. Yearly mean glycated hemoglobin (HbA1c) was 74 mmol/mol (8.9%). In December 2019, she was admitted to emergency room because of another severe hypoglycemia with loss of consciousness due to inappropriate insulin administration. After this event, patient started real-time continuous glucose monitoring (CGM—Medtronic Guardian Connect, Northridge California).

**Fig. 1:**
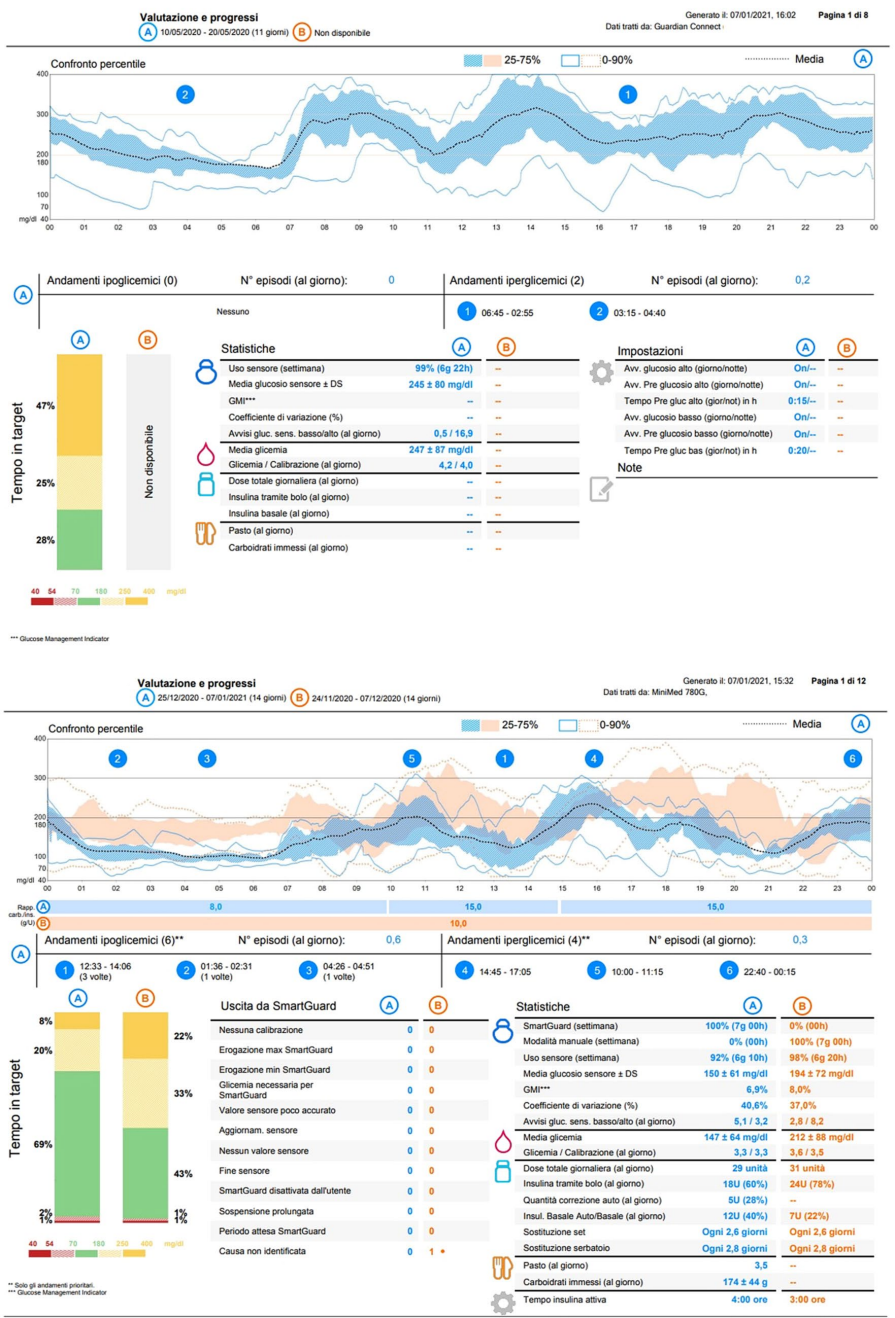
Subject’s continuous glucose monitoring (CGM) graphics on MDI (upper light blue) and on sensor-augmented pump therapy (Auto Mode in blue and Manual Mode in orange). On the bottom-left CGM ranges of period in Auto Mode (column A) and period in Manual Mode (column B)

The examination of CGM data let her more aware of her glycemic patterns and their high variability. Therefore, in November 2020, she accepted to start CSII (Medtronic MiniMed 780G, Northridge California). At time of CSII initiation, she was 1.68 m tall, her weight was 57 kg, with BMI 20.4 kg/m^2^. She did not present any signs of malabsorption, diarrhoea or weight loss. In conjunction with hypoglycemia, she was educated to eat 15 g of carbohydrates and to check glycemia after 15 min until she recovered from the episodes. After initial training, she started CSII on Manual Mode, switching to Auto Mode (AM) 15 days later, with glycemic target set on 110 mg/dL. Figure 1 resumes improvements of CGM metrics after 1 month in AM: there was an increase in time in range (TIR, 70–180 mg/dl) from 43 to 69%, almost achieving the goal suggested by the Advanced Technologies & Treatments for Diabetes (ATTD) Consensus on CGM; time above range (TAR) decreased from 55 to 28%, without increasing time below range, which does not reach the suggested 4% limit. No level 2 hypoglycemic episodes (< 54 mg/dl) were recorded [1]. These results could also be compared with previous CGM data, when she presented TIR 28% and TAR 72% (25% in level 1 and 47% in level 2). Improvement was also confirmed by HbA1c that decreased to 58 mmol/mol (7.5%). We can detect a slight increase in coefficient of variation, switching from 37% up to 40%. As notable in Fig. 1, in the first 2 weeks patient did not use bolus calculator but preferred to manually calculate boluses. The registered I:CHO was 1:10, as used on MDI. After the first 2 weeks, I:CHO was changed accordingly because of rapid decreasing glycemic after lunch and dinner, while increased for breakfast because of different quality of carbohydrates taken (higher glycemic index). We further changed the time of active insulin in the following weeks (Figure not available). Furthermore, in the CGM-only period she did not registered any other information rather than glycemic values.

In diabetes secondary to pancreatectomy, usually referred as T3cDM, insulin therapy management is highly challenging because of the high risk of severe hypoglycemic episodes. There are not specific guidelines, but targets used for T1DM and T2DM are recommended, although higher glycemic values are often considered acceptable. In advanced cases, insulin pumps should be considered, exploiting carbohydrate counting and trend arrows [2]. Type 1 diabetes and pancreatectomy could seem similar conditions because of insulin deficiency, but they are very different mainly because of the absence of glucagon as consequence of total pancreatectomy. Niwano et al. demonstrated that in subjects with pancreatectomy basal insulin and total daily insulin requirements are lower compared to subjects with type 1 diabetes. For example, subjects with total pancreatectomy do not require to increase insulin infusion rate early in the morning as seen in T1DM [3]. A recent case–control study evaluated throughout CGM glycemic variability in subjects with pancreatectomy confirming higher mean plasma glucose values and higher 60 min CONGA, while coefficient of variation and standard deviations were comparable with HbA1c-matched subjects with T1DM [4]. Notably all subjects included in this study were on multiple daily injections, 7/10 on detemir twice daily and 3/10 on glargine (both 100 UI/ml and 300 UI/ml). On MDI and CGM, our patient presented mean glucose value (247 mg/dl or 13.8 mmol/l) and standard deviation (87 mg/dl or 4.9 mmol/l) higher than those described by Juel et al. (respectively, 190 mg/dl or 10.6 mmol/l and 68 mg/dl or 3.8 mmol/l). We chose to take advantage of advanced hybrid closed loop (AHCL) to improve mean glucose levels and minimize variability: in fact, in subjects with T1DM AHCL increased of 12.5% TIR, with greater improvement overnight [5].

AHCL allowed our patient to improve metabolic control and all the glycemic variability metrics. Even more, it let patient to gain confidence with the over-all system and especially with lower glycemic values, reducing the fear of hypoglycemia. Patient reported improvement of quality of life and the reduction in diabetes burden. The improvement of quality of life was not assessed by any specific test, but it was evaluated during visit: patient referred less anxiety in the management of diabetes and to feel more secure with glycemic fluctuations. Improvement of metabolic control has been confirmed at follow-up visit, after 3 months, when patient presented 69% of TIR in the 14 days before the visit and HbA1c level was 7.2% (55 mmol/mol). Our case suggests how AHCL could be a successful approach in challenging subjects with T3cDM. In those subjects with long life-expectancy or good prognosis from an oncologic point of view after pancreatectomy, AHCL system, avoiding severe hypoglycemia and major hyperglycemic events and improving quality of life, should be considered also a cost-effective option. Further studies and clinical trial should be designed to confirm and support our observations described in this clinical case.

Patient agreed to publish her case report and to publish figures of her CGM anonymously. She signed informed consent.
